# Brazilian scale for evaluation of mental health care needs (CuidaSM): evidence of validity in primary health care

**DOI:** 10.1371/journal.pone.0323833

**Published:** 2025-07-10

**Authors:** Joana Moscoso Teixeira de Mendonça, Ana Alice Freire de Sousa, Ilana Eshriqui, Flávio Rebustini, Daiana Bonfim, Daniella Sampaio Zorzi, Camila Nascimento Monteiro, Talita Rewa, Leticia Yamawaka de Almeida

**Affiliations:** 1 Hospital Israelita Albert Einstein, São Paulo, São Paulo, Brazil; 2 Department of Gerontology, School of Arts, Sciences and Humanities (EACH), University of São Paulo, São Paulo, São Paulo, Brazil; Universidade Federal do Rio Grande do Norte, BRAZIL

## Abstract

**Background:**

In primary healthcare (PHC) settings, initiatives to identify mental health care needs (MHCN) are needed to ensure integral and quality care. However, there are few valid tools supporting health professionals in decision-making. This study aimed to obtain evidence of validity of the Brazilian Scale for Evaluation of Mental Health Care Needs (CuidaSM) in PHC.

**Methods:**

A group of health professional experts developed 130 dichotomous items, which were assessed by a heterogeneous group of panelists from different regions of Brazil (n = 73). Content validity ratio (CVR) was calculated, resulting in a second version of the scale with 43 items, which was administered to 879 healthcare users to provide evidence of internal-structure validity using exploratory factor analysis (EFA). Dimensionality was assessed through robust parallel analysis and the model was then cross-validated to create the final CuidaSM version. The CuidaSM score was subjected to normalization.

**Results:**

The final version of CuidaSM comprised 31 items, which were divided into two blocks: a “self-referred” block with 17 items distributed into five dimensions (Social Relationships, Functioning, Autonomy, Impulsiveness and Aggressiveness, and Spirituality) and a “health professional evaluation” block with 14 items and three dimensions (Violence, Self-aggression and Suicidal Behavior, and Caregiving Plan). The final model’s explained variance was 62.7%. Dimensionality values pointed to a multidimensional model (UNICO = 0.79; ECV = 0.69; and MIREAL = 0.22). All indicators were within adequate and satisfactory limits. CuidaSM score normalization stratified MHCN into four strata.

**Conclusions:**

The CuidaSM scale is a concise instrument combining healthcare user self-assessment and health professional clinical assessment. CuidaSM show satisfactory evidence of validity and was found to be a consistent, reliable, and robust instrument capable of accurately measuring MHCN in the PHC setting in Brazil.

## Introduction

Mental health disorders are among the top leading causes of burden of disease worldwide, with an important increase since 1990 [1]. However, the literature shows that the gap in the care provided to users presenting some sort of mental pain remains one of the major challenges for healthcare systems because most people with mental disorders do not receive the treatment they need [2–5].

Accordingly, the development of actions focused on consolidating and broadening the offer of mental healthcare services, as well as the timely access to resources is seen as priority worldwide [1–4]. It is essential that these actions comply with current recommendations for healthcare systems, which are grounded in propositions for community-based diversified services, including primary healthcare (PHC) and the person-centered care approach, while incorporating human rights principles [3–6].

In the Brazilian context, public policies have been implemented to integrate mental health care within PHC, promoting a more comprehensive, accessible, and coordinated approach. A major turning point occurred with the enactment of Law 10.216/2001 [7], which redefined the mental health care model by prioritizing the protection and rights of individuals with mental disorders.

This law replaced hospital-centered care with community-based services and created a network of substitute mechanisms to support recovery and social inclusion. Significant progress has been achieved within the Unified Health System (SUS) through these efforts, particularly with the establishment of the Psychosocial Care Network (RAPS) by Ordinance GM/MS 3.088/2011 [8], later consolidated in Ordinance 03/2017 [9]. RAPS emphasizes PHC as the central point for coordinating mental health services, ensuring that care is integrated, territory-based, and continuous.

Despite these advancements, challenges remain in aligning service availability with the growing demand and diverse needs of the population. This necessitates a more balanced regional distribution of community services and resources, improved coordination between Primary Health Care and specialized services, and the implementation of active strategies to combat stigma associated with mental disorders [10].

In this regard, two structuring elements must be acknowledged for the organization of mental health care: the first regards the allocation of resources given the somewhat limited budget scenario, whereas the second element refers to the logic of healthcare delivery, which must be guided by the psychosocial model to ensure autonomy, freedom, and the exercise of citizenship rights. In both cases, individuals’ needs are featured as the origin of all propositions.

In conceptual terms, one can identify different meanings and classifications for the term ‘need’ in the health literature; they are mainly used at service planning and provision scope [11–12].Regarding mental health, studies have leaned on discussing the perceived need of services to observe how people perceive their need of the type of care/treatment to be provided to them (or not), and factors related to it [13–22].

This corpus of evidence has been developed in high-income countries, based on different designs, populations, and data-collection techniques. However, there is a lack of tools to evaluate the dimensions surrounding the needs experienced by users to assist healthcare professionals in decision-making regarding the provision of the proper care, and the right service, while making the most efficient use of resources.

Considering the assumptions about the psychosocial model of mental health care [23–24], it is important to highlight that the concept of mental health care needs (MHCN) comprises multiple factors and is not limited to clinical features. Thus, this study aimed to obtain evidence of validity the Brazilian Scale for Evaluation of Mental Health Care Needs (CuidaSM) in PHC as an instrument capable of stratifying MHCN at the individual level.

## Methods

This psychometric study focused on the evaluation of evidence of content validity and internal structure, according to the American Educational Research Association (AERA), American Psychological Association (APA), and the National Council on Measurement in Education (NCME) (2014) [25]. Each of the study stages will be detailed in the following sections.

### Instrument preliminary stage

The development of the scale items took place through face-to-face workshop conducted by an expert Psychometry researcher, comprising 8 moments with a total of 60 hours. Workshops were conducted with a multidisciplinary group comprising 15 health professional experts from different health-related areas (Medicine, Psychology, Nursing, Nutrition, Dentistry, Pharmaceutics, Speech Therapy, and Public Health), all of whom with academic experience including specialization, master’s degree or PhD in PHC, public health, and/or mental health. Additionally, all professionals had practical experience and were working in clinical assistance and public health services management.

The psychometry expert started with an introduction of the theoretical background of the basic concepts of psychometrics and guided participants to develop the scale items based on four main practices: definition of the latent variable (defined as MHCN); literature search; writing the scale items while working in small groups; and discussion of proposed items about their clarity and pertinence to measure the latent variable with all participants. Thus, the workshop sessions included a technical and scientific foundation related to the application of various contemporary techniques in the search for validity evidence, including Content Validity, Response Process, Internal Structure, Evidence with Other Variables, and Consequences of Testing, in accordance with the most recent recommendations. These sessions were followed by practical activities and groups discussions, allowing the participating professionals to go through the stages of instrument development.

At this process, a literature search was conducted to identify the main theoretical references and instruments related to the latent variable (MHCN). Considering PHC attributes and Brazil’s Unified Health System (SUS) principles, the model of care for chronic conditions [26] was adopted as one of the references to support the rationale of CuidaSM. This model was proposed in the Brazilian context assuming the organization and integration of health services in healthcare networks based on the population health needs. The model of care for chronic conditions, developed based on three widely known models used worldwide (Chronic Care Model [CCM], Kaiser Permanente Pyramid Risk Model, and Dahlgren-Whitehead Social Model of Health), is represented by a pyramid that illustrates the stratification between self-care and the care provided by health professionals through a population-based health management approach. In the context of mental health care, such aspects can support planning interventions to prevent mental illness aimed at the population that already presents risk factors and/or management of cases of people with mental disorders according to worsening and complexity of the condition, similar to the approach of stepped-care protocols [27–28].

Considering the multifactorial character of MHCN, other references were adopted in addition to this theoretical framework. These were: psychosocial rehabilitation [23–24]; the International Classification of Functioning, Disability and Health (ICF) [29]; the Intervention Guide for mental, neurological and substance use disorders in non-specialized health settings: mental health Gap Action Programme (mhGAP-IG) [2]; and the World Health Organization Quality-of-Life Scale (WHOQOL-100) [30].

Guided by the references and instruments listed above, health professional experts wrote 130 dichotomous items (“no” or “yes”) comprising topics relating to the following multidimensional characteristics of the latent variable MHCN: social, autonomy, functioning, interpersonal relationships, spirituality, and clinical. The instrument was designed to be administered in two steps to cover both the individual’s perception about their needs and the professional’s evaluation.

### Stage 1: content validity evidence

The first version of the scale was assessed by a heterogeneous group of panelists who were recruited using the snowball technique [31]. Invitations were sent via WhatsApp and e-mail. This panel comprised 73 health professionals from different professions (nurses, psychologists, PHC physicians, psychiatrists, and other multidisciplinary health professionals) distributed within the five Brazilian regions (Southeast: 54.1%; South: 19.4%; Northeast: 15.2%; North: 5.5%; and Central-West: 5.5%). Most panelists were female (72.2%), mean age was 39.6 years (SD = 9.61 years), most had a graduate degree (84.7%) and worked in PHC services (31.9%), mental health specialist services (22.2%), PHC multidisciplinary team (6.9%), universities (6.9%), public health departments (16.6%), or others (16.6%).

After accepting to participate in the study and signing the Informed Consent Form (ICF), panelists were asked to answer two questionnaires: i) sociodemographic and professional profile and ii) questions relating to the items’ relevance and clarity, with checkboxes for multiple choices in addition to an open-ended question in case panelists wanted to add comments regarding the item. Data collection and management were completed using Research Electronic Data Capture (REDCap©) software [32].

The analyses provided by panelists were used to calculate the content validity ratio (CVR) [33] based on the number of panelists [34–35]. Considering that the number of panelists was 73, CVR was set at a critical value of 0.11. Thus, items ranking equal to or higher than the critical value were selected to the internal structure stage. CVR was applied to validate an item’s relevance, i.e., to assess whether the item effectively measures the latent variable (MHCN). CVR was represented by CVR-I (an item’s CVR) and CVR-E (the scale CVR), which is the average of the criteria’s CVR.

The current study followed a hierarchical flow because other indicators, such as item clarity, were only analyzed after the item had been considered relevant (i.e., item relevance) by some of the panelists. At this step, the application tends to inflate the mean recorded for CVR and to move to the next step the items that do not measure the latent variable. Accordingly, as pointed out by DeVellis (2017) [36], the item can be relevant, but the wording may be problematic. Thus, the second step referred to the items’ clarity, i.e., whether the items are semantically well-written. The third step aimed at assessing the need of changing the items’ phrasing. Mean CVR was only applied to items that measured the phenomenon.

### Stage 2: evidence of internal structure validity

The scale version reviewed by the group of panelists (CuidaSM, version 2) was administered to healthcare service users registered in 11 PHC services. It is important to note that PHC service selection was designed to include at least one unit from each geographic region of the country of heavily populated municipalities participating in the PlanificaSUS project [37] or other municipalities adopting the Health Care Planning method to implement the model of care for chronic conditions.

Data collection was conducted between November 2021 and August 2022, by a research team comprising health professionals who attended a 10-hour theoretical and practical training course, in PCH services from the five Brazilian regions: one service in the North (state of Roraima), one in the Northeast (Pernambuco), two PHC units in the Central-West (Mato Grosso), five in the Southeast (three in São Paulo and two in Minas Gerais), and two in the South (Paraná).

The selection of the states and municipalities for the data collection was based on a convenience sampling approach, which occurred after contacting management staff of the services to manage the schedule of activities. At this context, the lead researcher had virtual meetings with health professionals from each PHC service to invite them to contribute to the study and to systematize the flow of data collection.

The 11 PHC services were distributed at 7 municipalities (1 at North, 1 at Northeast, 1 at Central-West, 2 at Southeast and 2 at South). The choice of states and municipalities considered that the selected units were prepared and able to participate in the study at the time of data collection, but also the diversity in demographic and health profiles, and service availability, which can provide the different dynamics of PHC across the five Brazilian geographic regions (considering number of family health teams, population coverage, service structure). We clarify that two states at Southeast (São Paulo and Minas Gerais) were included considering the particularity of the city of Sao Paulo, which is the city with the biggest population of Brazil and has characteristics that are improbable to match another Brazilian context.

The inclusion criteria were: over 18 years and use of the PHC service for an individual consultation with a physician, nurse, or other health professional in the multidisciplinary team. Healthcare users who went to the service for emergency care, dental emergencies, or health procedures (vaccination, wound dressing changing, and medication administration) were excluded from the study.

Data collection was conducted in two steps: first, healthcare users were approached by researchers in the PHC service waiting room and invited to participate in the study. After reading and signing the ICF, participants were asked to answer questions assessing their sociodemographic and clinical profile, in addition to part of the CuidaSM scale. Data were registered by researchers in tablet computers using the REDCap platform.

After completing the questionnaire, participants were identified with a colored paper bracelet to ensure data-collection continuity. Participants were then asked to inform about their participation in the study at the beginning of the consultation with the health professional they were waiting to see. The second moment of the data-collection procedure comprised the clinical assessment of healthcare users, when 32 health professionals of 13 PHC teams or multiprofessional teams were invited to collaborate to the study by conducting a clinical assessment of participants based on a printed version of the scale for professionals — questionnaires were collected by the research team at the end of the day and data were entered into the REDCap platform.

Initially, 1,219 healthcare users accepted to participate in the study, but those who did not complete the health professional evaluation phase were excluded from the analysis. Thus, the final sample of the study comprised 879 health care users. This sample (total and/or per region) reflected the volume of users that attend the PHC services at different Brazilian contexts during the data collection period and had an individual consultation with a graduated PHC professional, showing consistency with the population density of the Brazilian regions (Southeast has the greatest one).

### Data analysis

#### Exploratory factor analysis (EFA).

Exploratory factor analysis (EFA) was used to test the internal structure of the scale. The first fundamental stage consisted of assessing whether data could be factorial using a measure of sampling adequacy (MSA) statistic. Bartlett’s test of sphericity, the determinant of the matrix, and the Kaiser–Meyer–Olkin (KMO) test were also conducted at this stage. In addition to database evaluation, individual items were assessed separately based on recommendations by Lorenzo-Seva and Ferrando (2021) [38] because items inappropriate for factor analysis could affect model solution. The missing values were handled using the multiple imputation technique [39].

Dimension testing was performed using parallel analysis (PA) based on optimal implementation of parallel analysis with minimum rank factor analysis, which minimizes common residual variance [40] as inferred by tetrachoric correlation. PA is one of the most robust and accurate techniques for testing the dimensions underlying a set of data [41–44] and was implemented through permutation with 500 random matrices. Factor extraction was performed using the unweighted least-squares (ULS) technique, which reduces the matrices’ residues [45]. A promin (oblique) rotation was used if the instrument emerged as multidimensional [46].

One-dimensional congruence (UNICO > 0.95), explained common variance (ECV > 0.80) [47], and mean of item residual absolute loadings (MIREAL < 0.30) were used as indicators for one-dimensionality [48].

#### Instrument quality parameters.

The instrument’s explained variance must be close to 60% [49]. Initial factorial loads of 0.30 are recommended when the sample comprises less than 300 individuals [49] and the communities must register values higher than 0.40 [50]. Item maintenance in, or removal from, the model will depend on the magnitude of factorial loads, communalities, the existence of cross loading, Heywood cases, and the interpretability of factors. To increase decision-making accuracy of item maintenance or removal, we determined the unique directional correlation (eta) between a given factor and an item using Pratt’s measure [51].

#### Goodness-of-fit index.

Parameters adopted for goodness-of-fit cutoff were based on the study by Sivo et al. (2006) [52]. Minimum indices for adequacy were nonnormed fit index (NNFI ≥ 0.97), goodness-of-fit index (GFI ≥ 0.93), adjusted goodness-of-fit index (AGFI ≥ 0.91), and root mean square of residuals (RMSR ≤ 0.10); comparative fit index (CFI ≥ 0.97) and root mean square error of approximation (RMSEA ≤ 0.07) were also used. The ULS estimation was used.

#### Reliability.

Reliability was assessed based on four indicators: Cronbach’s alpha (α) [53], greatest lower bound (GLB) [54], McDonald’s omega (ω) [55] — all computed through Bayesian estimation, and Overall Reliability of Fully-Informative prior Oblique N-EAP (ORION) scores [56].

### Stage 3: score standardization

Initially, an exploratory descriptive analysis of MHCN general scores was conducted. Items and total scores results were represented by the frequency of answers, median (Md), interquartile range (IQR), amplitude (amp), minimum (min), and maximum (max) values.

A regularized linear regression with elastic-net regularization using total score as the dependent variable and items as predictors was calculated to identify the instrument items that can serve as relevant markers for MHCN. The elastic net was used to evaluate the weight of the items in the composition of the final score and, therefore, to assess their impact on mental health. The holdout technique was used for cross-validation — 50% of the sample for the training set and 50% for the test set. One of the assumptions for the predictor variables in regularized regression is that they must be independent; however, the items are correlated and, possibly, strongly correlated. For this reason, we chose elastic-net regularization, which handles well strongly correlated items [57].

The first standardization stage consisted of establishing score cutoffs based on the frequency distribution of participants’ answers. Although this is a common process in standardization studies, it can lead to distortions, because the scores are not actually analyzed, but taken as a consequence of the participants’ position in the cutoff points. Discriminant analysis of MHCN limits and scores was used to achieve a higher accuracy in determining the score limits and to assess the ability of the classification to predict the individuals’ MHCN. Discriminant analysis is used to separate two or more groups of observations and to predict the likelihood of an entity (individual or object) to remain in a given class or specific group based on the various independent variables [58], determining which independent variables contribute most to the differences in the mean score profiles of the groups [58]. Thus, it is possible to confirm whether the cutoff values established by the distribution have the ability to appropriately classify the individuals within the MHCN limits.

All analyses were performed using Factor 12.01.01 (Ivanti, South Jordan, UT, USA), IBM SPSS Statistics for Windows v.23 (IBM Corp., Armonk, NY, USA), and JASP 16.04 (JASP Team) software.

### Ethics approval

The study was approved by the Hospital Israelita Albert Einstein Ethics Committee (HIAE/CAAE: 12395919.0.0000.0071). Participation was conditional on the completion and signing of the Informed Consent Form.

## Results

### Stage 1: content validity evidence

The group of health professional experts developed the first version of CuidaSM comprising 130 items, which were submitted to analysis by the group of panelists and CVR calculation, which retained 43 of the 130 items that emerged as relevant initial propositions. All relevant items also had clarity values higher than the CVR critical value for the item. Table 1 presents the CVR scores for items retained in the preliminary version of the scale.

**Table 1:** Content validity ratio (CVR) scores for items retained in the preliminary version of the CuidaSM scale.

Item	Relevance	Clarity
16. Do you have family support?	0.17	0.78
23. Do you have friends?	0.14	0.75
24. Do you talk to your friends?	0.11	0.64
35 Are you able to go to healthcare services by yourself?	0.11	0.72
36. Whenever you need, can you take your medication at the times scheduled by the doctor?	0.11	0.67
37. Are you able to perform your work tasks?	0.17	0.56
38. Are you able to keep working?	0.11	0.53
40. Can you catch up with your school assignments?	0.14	0.58
42. Are you able to bath by yourself?	0.14	0.75
43. Do you perform your daily hygiene by yourself?	0.17	0.72
44. Do you get dressed by yourself?	0.14	0.75
47. Are you able to shop for your daily supplies by yourself?	0.11	0.69
51. Can you socially interact with people?	0.17	0.56
56. Are you able to keep friendships?	0.14	0.78
58. Are you able to control your impulsiveness?	0.17	0.61
59. Are you able to control your verbal aggressiveness?	0.22	0.64
60. Are you able to control your physical aggressiveness?	0.19	0.56
63. Do you have good family relationships?	0.14	0.67
68. Do you perform any leisure activity?	0.11	0.69
77. Do you find a meaning for your life?	0.11	0.67
78. Do you feel that your life has a purpose?	0.11	0.69
79. Can you admire things around you?	0.14	0.61
83. Are you hopeful with your life?	0.11	0.64
89. Was the user a witness of violence?	0.22	0.56
90. Was the user an author of violence?	0.19	0.47
91. Was the user a victim of violence?	0.25	0.47
92. Does the user have a clinical profile compatible to depressive syndrome?	0.25	0.44
93. Does the user have a clinical profile compatible to psychotic syndrome?	0.22	0.39
94. Is the user an elderly with dementia signs?	0.25	0.36
95. Does the user present signs of disorder due to alcohol abuse?	0.19	0.42
96. Does the user present signs of disorder due to drug abuse?	0.19	0.42
97. Does the user have a will to die?	0.14	0.42
98. Does the user have suicidal ideas?	0.19	0.42
99. Does the user plan to commit suicide?	0.19	0.44
100. Has the user attempted suicide?	0.19	0.47
101. Does the user think about self-aggression?	0.17	0.44
102. Does the user present imminent risk of self-aggression?	0.17	0.33
103 Does the user have history of self-aggression?	0.17	0.44
104. Does the user present psychomotor agitation?	0.19	0.39
105. Does the PHC team have a hard time handling this case?	0.14	0.39
106. Does the user deny their disease?	0.17	0.44
107. Is the user unaware of their condition?	0.11	0.44
108. Does the user resist the proposed caregiving plan?	0.22	0.42

### Stage 2: internal validation evidence

Our final sample of 879 healthcare users who participated in this stage of study had a mean age of 45.0 years (± SD = 16.7 years). Table 2 presents the socioeconomic profile of study participants.

**Table 2:** Socioeconomic profile of survey participants.

Variable	N	%
**Sex**	833	
Female	619	74.3
Male	214	25.7
**State**	880	
São Paulo	461	52.4
Minas Gerais	141	16.0
Pernambuco	35	4.0
Roraima	48	5.4
Mato Grosso	97	11.0
Paraná	98	11.1
**Schooling**	824	
< 5 years	18	2.2
≥ 5 years and < 9 years	359	43.6
≥ 9 years and < 12 years	351	42.6
≥ 12 years	96	11.6
**Marital status**	824	
Single	300	36.4
Married/common-law marriage	383	46.5
Widow/widower	63	7.6
Separated/divorced	44	5.3
Other	34	4.1
**Social program beneficiary**	821	
No	624	76.0
Yes	197	24.0
**Sanitation system in the house**	808	
Sewage system	695	86.0
Other (cesspool or without sewage system)	113	14.0
**Electric power available in the house**	818	
No	17	2.1
Yes	801	97.9

The instrument was administered in the field; it included 23 items in the “self-referred” block and 20 items in the block that represented “health professional evaluation”. The first step of this process involved evaluating sample adjustment measures aimed at assessing database factorability. Three items in the “self-referred” block did not show adequate factorability and were removed from the analysis based on recommendations by Lorenzo-Seva and Ferrando (2021) [38]. The analysis was performed with the 40 remaining items. These items’ MSA indicated good factorability as measured by the KMO statistic (0.73), Bartlett’s test of sphericity (6,568.1, df = 1,891; p < 0.0001), and the determinant of the matrix (< 0.000001).

Initial PA had 40 items and indicated the possibility of including 11 dimensions. Thus, the analysis was performed by establishing the 11-dimension model as the initial configuration. The analysis showed several items with adjustment issues, leading to the successive removal of items for adjusting the model to the two model principles: quantitative and interpretative. The selection of items to be removed considered the set of primary indicators: factorial loads, communicability, Eta for Pratt’s importance measure, and model adjustment indices. This process resulted in changes in the number of instrument dimensions — the model was adjusted to include eight dimensions after 19 items were removed from the initial model. This model with 40 items remained with adequate factorability as shown by the KMO statistic (0.75), Bartlett’s sphericity (8,425.4, df = 903; p < 0.0001), and matrix determinant (< 0.000001). This model’s explained variance was 62.7%.

Closeness to undimensionality assessment values pointed to a multidimensional model: UNICO = 0.79, ECV = 0.69, and MIREAL = 0.22. Table 3 shows the values for the dimensionality indicators of the 31 items retained in the model.

**Table 3:** Values for dimensionality indicators (I-UNICO, I-ECV, and I-Real) of the items retained in the model.

	Variable	I-UNICO	I-ECV	I-REAL
V1	Do you have friends?	0.828	0.596	0.329
V2	Do you talk to your friends?	0.895	0.667	0.308
V4	Are you able to go to healthcare services by yourself?	0.990	0.874	0.115
V5	Are you able to perform your work tasks?	0.959	0.773	0.229
V6	Are you able to keep working?	0.965	0.787	0.219
V8	Are you able to bath by yourself?	0.018	0.017	0.655
V9	Do you perform your daily hygiene by yourself?	0.004	0.004	0.741
V10	Can you get dressed by yourself?	0.025	0.024	0.591
V7	Are you able to shop for your daily supplies by yourself?	0.866	0.634	0.332
V3	Are you able to keep friendships?	0.998	0.940	0.077
V11	Are you able to control your impulsiveness?	0.999	0.969	0.066
V12	Are you able to control your verbal aggressiveness?	1.000	0.979	0.066
V13	Are you able to control your physical aggressiveness?	0.997	0.927	0.109
V14	Can you find a meaning for your life?	0.989	0.872	0.177
V15	Do you feel that your life has a purpose?	0.988	0.862	0.181
V16	Can you admire things around you?	0.994	0.898	0.104
V17	Are you hopeful with your life?	0.991	0.878	0.142
V18	Was the user a witness of violence?	0.888	0.659	0.167
V19	Was the user an author of violence?	0.975	0.815	0.105
V20	Was the user a victim of violence?	0.975	0.813	0.118
V21	Does the user have a will to die?	0.997	0.934	0.140
V22	Does the use have suicidal ideas?	0.985	0.849	0.225
V23	Does the user have suicidal plans?	0.990	0.877	0.186
V24	Has the user attempted suicide?	0.967	0.792	0.291
V25	Does the user think about self-aggression?	0.960	0.774	0.272
V26	Does the user present imminent risk of self-aggression?	0.980	0.830	0.222
V27	Does the user have history of self-aggression?	0.963	0.781	0.255
V28	Does the PHC team have a hard time handling this case?	0.998	0.940	0.111
V29	Does the user deny their disease?	0.999	0.948	0.121
V30	Is the user unaware of their condition?	0.986	0.854	0.168
V31	Does the user resist the proposed caregiving plan?	0.998	0.944	0.120

I-UNICO: one-dimensional congruence for the item; I-ECV: explained common variance for the item; and I-Real: residual absolute loading for the item.

The final model (Table 4) included five dimensions for the “self-referred” block, which comprised 17 items: three items for “social relationships”, four items for “functioning”, three items each for “autonomy” and “impulsiveness and aggressiveness”, and four items for “spirituality”. The “health professional evaluation” block comprised 14 items divided into three dimensions: “violence” (three items), “self-aggression and suicidal behavior” (seven items), and “caregiving plan” (four items). Additional file 1 presents the Portuguese version of CuidaSM. Factorial loads for the “self-referred” block ranged from 0.40 to 0.98, communicability ranged from 0.15 to 0.94, and Pratt’s measure values ranged from 0.45 to 0.97, whereas factorial loads for the “health professional evaluation” block ranged from 0.38 to 0.85, communicability ranged from 0.26 to 0.59, and Eta for Pratt’s measure ranged from 0.41 to 0.84. There were no Heywood cases, cross-loading, or collinearity/multicollinearity issues capable of suggesting item redundancy and overlapping.

**Table 4:** Factorial loads, communicability, and Pratt’s measure for the final model.

			Item	Factorial load								*h* ^*2*^		Pratt’s measure (ETA)							
				D1	D2	D3	D4	D5	D6	D7	D8		D1	D2	D3	D4	D5	D6	D7	D8	
Self-referred domain	Social relationships	V1	Do you have friends?	**0.854**	−0.080	0.017	−0.025	0.086	0.048	0.094	−0.101	*0.738*		**0.834**	0.000	0.034	0.000	0.152	0.000	0.000	0.133
		V2	Do you talk to your friends?	**0.912**	−0.053	−0.007	−0.043	0.107	0.024	0.038	−0.027	*0.838*		**0.895**	0.000	0.000	0.000	0.182	0.000	0.000	0.061
		V3	Are you able to keep friendships?	**0.345**	0.084	−0.032	0.133	−0.117	0.012	−0.153	0.095	*0.200*		**0.353**	0.120	0.000	0.173	0.000	0.000	0.175	0.000
	Functioning	V4	Are you able to go to healthcare services by yourself?	0.115	**0.319**	−0.003	0.053	−0.055	−0.024	−0.053	0.003	*0.157*		0.151	**0.337**	0.000	0.095	0.000	0.049	0.097	0.000
		V5	Are you able to perform your work tasks?	−0.060	**0.814**	−0.029	−0.035	0.057	−0.004	0.044	0.001	*0.623*		0.000	**0.783**	0.000	0.000	0.099	0.021	0.000	0.000
		V6	Are you able to keep working?	−0.009	**0.853**	−0.050	−0.047	0.015	0.072	0.016	0.002	*0.675*		0.000	**0.820**	0.000	0.000	0.047	0.000	0.000	0.000
		V7	Are you able to shop for your daily supplies by yourself?	0.089	**0.407**	0.189	0.043	0.048	−0.038	−0.003	−0.085	*0.328*		0.147	**0.458**	0.226	0.098	0.095	0.071	0.026	0.145
	Autonomy	V8	Are you able to bath by yourself?	0.095	0.005	**0.741**	−0.079	0.073	−0.002	0.006	0.039	*0.582*		0.114	0.027	**0.746**	0.064	0.076	0.002	0.000	0.028
		V9	Do you perform your daily hygiene by yourself?	−0.041	−0.068	**0.986**	0.024	−0.021	0.012	−0.032	0.020	*0.946*		0.000	0.000	**0.972**	0.000	0.013	0.015	0.000	0.014
		V10	Do you get dressed by yourself?	−0.065	0.099	**0.726**	0.047	−0.069	0.003	0.024	−0.049	*0.562*		0.000	0.147	**0.731**	0.022	0.047	0.000	0.000	0.062
	Impulsiveness and aggressiveness	V11	Are you able to control your impulsiveness?	0.006	−0.037	0.009	**0.596**	0.069	−0.041	−0.030	0.064	*0.385*		0.036	0.000	0.000	**0.594**	0.140	0.070	0.075	0.000
		V12	Are you able to control your verbal aggressiveness?	−0.012	0.012	−0.005	**0.866**	−0.056	0.017	−0.046	0.084	*0.699*		0.000	0.050	0.012	**0.828**	0.000	0.000	0.103	0.000
		V13	Are you able to control your physical aggressiveness?	0.031	−0.039	−0.003	**0.587**	0.081	0.024	0.148	−0.189	*0.430*		0.084	0.000	0.007	**0.590**	0.149	0.000	0.000	0.229
	Spirituality	V14	Do you find a meaning for your life?	0.035	0.009	−0.007	0.084	**0.718**	0.034	0.007	−0.022	*0.587*		0.095	0.041	0.000	0.176	**0.737**	0.000	0.000	0.055
		V15	Do you feel that your life has a purpose?	0.074	0.098	−0.032	0.039	**0.619**	−0.001	−0.008	0.035	*0.468*		0.141	0.150	0.000	0.111	**0.642**	0.010	0.042	0.000
		V16	Can you admire things around you?	−0.070	−0.058	−0.001	−0.049	**0.719**	−0.026	0.019	−0.033	*0.462*		0.000	0.000	0.000	0.000	**0.677**	0.036	0.000	0.048
		V17	Are you hopeful with your life?	0.065	0.073	0.017	−0.019	**0.494**	−0.106	−0.065	0.084	*0.319*		0.119	0.118	0.027	0.000	**0.508**	0.132	0.121	0.000
Healthcare professional evaluation dimension	Violence	V18	Was the user a witness of violence?	0.025	0.044	0.021	0.027	−0.049	**0.665**	0.080	−0.038	*0.463*		0.000	0.000	0.025	0.000	0.065	**0.664**	0.132	0.000
		V19	Was the user an author of violence?	−0.038	0.019	0.029	−0.012	−0.008	**0.545**	−0.085	0.140	*0.308*		0.061	0.000	0.028	0.035	0.019	**0.531**	0.000	0.143
		V20	Was the user a victim of violence?	0.023	−0.002	−0.028	−0.006	−0.009	**0.770**	−0.002	−0.020	*0.590*		0.000	0.016	0.026	0.024	0.022	**0.767**	0.000	0.000
	Self-aggression and suicidal behavior	V21	Does the user have a will to die?	0.033	−0.023	−0.069	0.034	−0.114	0.033	**0.586**	−0.037	*0.381*		0.000	0.069	0.070	0.000	0.168	0.076	**0.581**	0.000
		V22	Does the user have suicidal ideas?	−0.117	0.052	0.014	0.083	−0.025	−0.039	**0.706**	−0.029	*0.478*		0.158	0.000	0.012	0.000	0.070	0.000	**0.670**	0.000
		V23	Does the user have suicidal plans?	−0.150	−0.036	−0.003	0.013	0.195	−0.030	**0.534**	0.134	*0.385*		0.186	0.090	0.008	0.000	0.000	0.000	**0.544**	0.215
		V24	Has the user attempted suicide?	−0.022	−0.083	0.024	−0.057	0.165	0.181	**0.636**	−0.046	*0.504*		0.059	0.149	0.017	0.109	0.000	0.247	**0.636**	0.000
		V25	Does the user think about self−aggression?	0.063	0.074	−0.003	0.003	−0.061	−0.048	**0.657**	0.030	*0.418*		0.000	0.000	0.000	0.000	0.111	0.000	**0.631**	0.086
		V26	Does the user present imminent risk of self−aggression?	0.112	0.076	−0.005	−0.027	−0.171	−0.101	**0.528**	0.105	*0.359*		0.000	0.000	0.000	0.071	0.217	0.000	**0.527**	0.171
		V27	Does the user have history of self−aggression?	0.062	−0.016	0.023	−0.018	−0.020	0.160	**0.525**	−0.005	*0.348*		0.000	0.056	0.023	0.056	0.058	0.215	**0.540**	0.000
	Caregiving plan	V28	Does the PHC team have a hard time handling this case?	0.042	−0.091	0.046	−0.071	−0.036	−0.007	0.076	**0.440**	*0.290*		0.000	0.151	0.033	0.128	0.071	0.000	0.150	**0.472**
		V29	Does the user deny their disease?	−0.006	0.035	−0.021	−0.015	0.024	−0.043	0.022	**0.856**	*0.732*		0.030	0.000	0.027	0.058	0.000	0.000	0.086	**0.848**
		V30	Is the user unaware of their condition?	0.075	−0.024	0.029	0.019	−0.059	−0.038	0.219	**0.382**	*0.261*		0.000	0.065	0.024	0.000	0.089	0.000	0.278	**0.414**
		V31	Does the user show resistance to the proposed caregiving plan?	−0.038	−0.013	0.000	0.042	0.047	0.103	−0.073	**0.869**	*0.723*		0.075	0.056	0.000	0.000	0.000	0.121	0.000	**0.837**

Goodness-of-fit indices showed adequate levels: NNFI = 0.97, CFI = 0.98, BIC = 2,249.18, GFI = 0.98, AGFI = 0.96, RMSEA = 0.03, and RMSR = 0.03. Accordingly, reliability indicators based on the Bayesian approach also had good levels: Cronbach’s alpha = 0.82 [95%CI: 0.80–0.83], McDonald’s omega = 0.80 [95%CI: 0.78–0.82], and GLB = 0.93 [95%CI: 0.92–0.94]. ORION values for the dimensions ranged from 0.79 to 0.95. Factorial solution-quality indices were satisfactory for factor determinacy index (FDI), sensitivity ratio (SR), and expected percentage of true differences (EPTD). Table 5 shows the synthesis of the final model for all indicators.

**Table 5:** Synthesis of final model.

	Index	Technique	FINAL MODEL
Exploratory	Adequacy of correlation matrix	Determinant of the matrix	< 0.000001
		Bartlett’s sphericity	8,471.6 (df = 465)
		KMO (Kaiser–Meyer–Olkin) test	0.74
	Explained variance (PA)		62.7%
	Correlation (r =)		−0.24 to 0.81
Reliability	Cronbach’s alpha (α)		0.82 [95%CI: 0.80–0.83]
	McDonald’s omega (ω)		0.80 [95%CI: 0.78–0.82]
	Greatest lower bound (GLB)		0.93 [95%CI: 0.92–0.94]
	ORION*		0.80; 0.79; 0.82; 0.86; 0.83; 0.75; 0.95; 0.89
Goodness-of-fit	Nonnormed fit index (NNFI)		0.97
	Comparative fit index (CFI)		0.98
	Schwarz’s Bayesian information criterion (BIC)		2249.18
	Goodness-of-fit index (GFI)		0.98
	Adjusted goodness-of-fit index (AGFI)		0.96
	Root mean square error of approximation (RMSEA)		0.03
	Root mean square of residuals (RMSR)		0.03
One-dimensional assessment	One-dimensional congruence (UNICO)		0.876
	Explained common variance (ECV)		0.684
	Mean of item residual absolute loading (MIREAL)		0.224
Quality and Effectiveness	Factor determinacy index (FDI)*		0.89; 0.89; 0.90; 0.93; 0.91; 0.86; 0.97; 0.94
	Sensitivity ratio (SR)*		2.02; 1.96; 2.16; 2.52; 2.22; 1.73; 4.49; 2.85
	Expected percentage of true differences (% EPTD)*		89.5; 88.5; 90.9; 90.9; 89.7; 87.2; 95.5; 92.1%

### Stage 3: score standardization

The instrument score must be interpreted; for the CuidaSM scale, the higher the instrument score, the greater the MHCN. The “self-referred” domain (block 1) comprises 17 items distributed into five dimensions. A score of zero (all ‘no’ answers) indicates a greater need of care. The “health professional evaluation” domain (block 2) comprises 14 items distributed into three dimensions and, contrary to block 1, a score of one (1) indicates a greater need of care. Thus, instead of inverting the score of the two domains, we developed a formula to calculate the instrument’s total score. The calculation was as follows: block 1 score = 17 (number of items in the domain) − number of answers ‘1’ (i.e., ‘yes’); thus, block 1 = (17 − Σblock 1) and block 2 is the sum of answers. The instrument total score is computed as follows:


$$\begin{array}{c} \text{CuidaSM}=\left(17-\Sigma \text{block}1\right)+\Sigma \text{block}2 \end{array}$$

The instrument may range from zero (lowest MHCN) to 31 (highest MHCN). It is worth noting that items in block 1 can be interpreted as MHCN reducers and items in block 2 as MHCN boosters.

Table 6 shows the frequency of answers given to questionnaire items. There is a clear prevalence of ‘yes’ answers for all items in block 1. Nevertheless, two items stood out for their frequency of ‘no’ answers: item 6, “Are you able to keep working?”, had the highest frequency of ‘no’ answers (17.6%) among all items; and item 11, “Are you able to control your impulsiveness?”, also had a higher frequency (16.4%) of ‘no’ answers. Contrary to block 1, the frequency of ‘no’ answers was higher than that of ‘yes’ answers for all block 2 items. However, two items in the “violence” dimension with a high frequency of ‘yes’ answers were indicative of the need for care: item 18, “Was the user a witness of violence?” (23.2%) and item 20, “Was the user a victim of violence?” (18.3%).

**Table 6:** Frequency of answers to CuidaSM scale items.

			Question/Score	Frequency of answers to the item (N/%)	
No	Yes
Self-referred domain	Social relationships	V1	Do you have friends?	88 (10.00)	795 (90.00)
		V2	Do you talk to your friends?	111 (12.60)	772 (87.40)
		V3	Are you able to keep friendships?	47 (5.30)	836 (94.70)
	Functioning	V4	Are you able to go to healthcare services by yourself?	40 (4.50)	843 (95.50)
		V5	Are you able to perform your work tasks?	113 (12.80)	770 (87.20)
		V6	Are you able to keep working?	155 (17.60)	728 (84.40)
		V7	Are you able to shop for your daily supplies by yourself?	63 (7.1)	820 (92.90)
	Autonomy	V8	Are you able to bath by yourself?	14 (1.60)	869 (98.40)
		V9	Do you perform your daily hygiene yourself?	11 (1.20)	872 (98.8)
		V10	Do you get dressed by yourself?	17 (1.90)	866 (98.10)
	Impulsiveness and aggressiveness	V11	Are you able to control your impulsiveness?	145 (16.40)	738 (83.60)
		V12	Are you able to control your verbal aggressiveness?	113 (12.80)	770 (87.20)
		V13	Are you able to control your physical aggressiveness?	49 (5.50)	834 (94.50)
	Spirituality	V14	Do you find a meaning for your life?	103 (11.70)	780 (88.30)
		V15	Do you feel that your life has a purpose?	94 (10.60)	789 (89.40)
		V16	Can you admire things around you?	47 (5.30)	836 (94.70)
		V17	Are you hopeful with your life?	115 (13)	768 (87.00)
Health professional evaluation domain	Violence	V18	Was the user a witness of violence?	677 (76.70)	206 (23.30)
		V19	Was the user an author of violence?	828 (93.80)	55 (6.20)
		V20	Was the user a victim of violence?	721 (81.70)	162 (18.30)
	Self-aggression and suicidal behavior	V21	Does the user have a will to die?	844 (95.60)	39 (4.40)
		V22	Does the user have suicidal ideas?	855 (96.80)	28 (2.30)
		V23	Does the user have suicidal plans?	863 (97.70)	20 (2.30)
		V24	Has the user attempted suicide?	832 (94,20)	51 (5.80)
		V25	Does the user think of self-aggression?	868 (98.30)	15 (1.70)
		V26	Does the user present imminent risk of self-aggression?	869 (98.40)	14 (1.60)
		V27	Does the user have history of self-aggression?	854 (96.70)	29 (3.30)
	Caregiving plan	V28	Does the PHC team have a hard time handling this case?	855 (96.80)	28 (3.20)
		V29	Does the user deny their disease?	871 (98.60)	12 (1.40)
		V30	Is the user unaware of their condition?	864 (97.80)	19 (2.20)
		V31	Does the user resist the proposed caregiving plan?	866 (98.10)	17 (1.9)

Scores exhibited great differences among MHCN strata because many scores had values close to the minimum limit; scores got farther from the median, which, in this case, was closer to the minimum value when scores were in the upper quartile. Accordingly, three initial classifications were suggested. The first classification model had three MHCN strata with the first cutoff at percentile 75; the second stratum ranged from percentile 76–90, and the third MHCN stratum started at percentile > 91. The second model included four MHCN strata: up to percentile 50, percentile 51–75, percentile 76–90, and percentile 91 + . The third model tested also had four strata: up to percentile 75, percentile 76–90, percentile 91–95, and percentile > 95.

Results for discriminant analysis of model 1 were MBox = 469.99, p < 0.001; λ_wilks_ = 0.24; F_(2, 880)_ = 1,371.94, p < 0.001; and canonical correlation = 0.87. Model 2 results for discriminant analysis were: MBox = 866.61, p < 0.001; λ_wilks_ = 0.109; F_(2, 879)_ = 2,386.15, p < 0.001; and canonical correlation = 0.944. In addition, model 2 was able to adequately classify 97.8% of the cases. Discriminant analysis results for model 3 were: MBox = 109.50, p < 0.001; λ_wilks_ = 0.13; F_(2, 879)_ = 1,843.95, p < 0.001; and canonical correlation = 0.929. This model adequately classified 88.4% of original cases. Thus, model 2 was the most adequate to establish the cutoffs for the MHCN classification limits. Table 7 presents the MHCN classification strata, percentile limits, and scores for the CuidaSM instrument.

**Table 7:** CuidaSM scale scores’ limits, classification, and interpretation.

MHCN stratum	Percentile limit	Score
Low MHCN	Up to 50	0 and 1
Moderate MHCN	51–75	2 and 3
High MHCN	76–90	4–6
Very high MHCN	90+	7+

MHCN: mental health care needs.

After establishing the instrument’s classification and interpretation limits, it is essential to better understand how the items influenced the instrument’s total score and identify the items capable of having a stronger influence on score formation. Thus, we computed a regularized linear regression with the elastic-net algorithm using the instrument’s total score as dependent variable whereas the items were used as predictors. Regression λ was 0.01 with mean standard error (MSE) of 0.002 and 0.003 in the training database and test database, respectively. Model R^2^ was 99.70% with mean absolute percentage error (MAPE) of 6.49% and root mean squared error (RMSE) of 0.06. Table 8 shows the β coefficients of instrument items. The results show that items with the highest frequency of answers opposite to the trend of answers in the same answer block had β values higher than 0.12. Thus, items 18 and 20 were those with the highest potential for MHCN, whereas items 6 and 11 had the highest potential for reducing the instrument’s scores in a more relevant way and could be interpreted as MHCN reducers.

**Table 8:** Beta (β) coefficients of CuidaSM scale items.

Item	Coefficient (β)
V1. Do you have friends?	−0.0913
V2. Do you talk to your friends?	−0.1112
V3. Are you able to keep friendships?	−0.0712
V4. Are you able to go to healthcare services by yourself?	−0.0632
V5. Are you able to perform your work tasks?	−0.1102
**V6. Are you able to keep working?**	*−0.1204*
V7. Are you able to shop for your daily supplies by yourself?	−0.0922
V8. Are you able to bath by yourself?	−0.0451
V9. Do you perform your daily hygiene by yourself?	−0.0368
V10. Do you get dressed by yourself?	−0.0257
**V11. Are you able to control your impulsiveness?**	*−0.1205*
V12. Are you able to control your verbal aggressiveness?	−0.1037
V13. Are you able to control your physical aggressiveness?	−0.0702
V14. Do you find a meaning for your life?	−0.1004
V15. Can you feel that your life has a purpose?	−0.1047
V16. Can you admire things around you?	−0.0687
V17. Are you hopeful for your life?	−0.1066
**V18. Was the user a witness of violence?**	*0.1291*
V19. Was the user an author of violence?	0.0818
**V20. Was the user a victim of violence?**	*0.1269*
V21. Does the user have a will to die?	0.0682
V22. Does the user have suicidal ideas?	0.0598
V23. Does the user have suicidal plans?	0.0513
V24. Has the user attempted suicide?	0.0766
V25. Does the user think about self-aggression?	0.0375
V26. Does the user present imminent risk of self-aggression?	0.0194
V27. Does the user have history of self-aggression?	0.0586
V28. Does the PHC team have a hard time handling this case?	0.0523
V29. Does the user deny their disease?	0.0294
V30. Is the user unaware of their condition?	0.0553
V31. Does the user resist the proposed caregiving plan?	0.0474

When analyzing the answers provided, especially those to items 18 and 20 (block 2) and 6 and 11 (block 1), we noticed a change in behavior, as the MHCN priority stratum increases. For example, 51.9% of participants who were classified in stratum 4 (very high MHCN) seem to have witnessed violence (item 18) and 49.4% were victims of violence (item 20); as for stratum 1 (low MHCN), these limits were 9.4% and 4.1%, respectively. As expected, answers in block 1 exhibited the opposite trend: 95.3% of participants reported to be able to keep working (item 6, stratum 1) and 95.5% reported to be able to control their impulsiveness (item 11, stratum 1). As for stratum 4, the frequency of answers was 44.4% for item 6 (work) and 39.5% for item 11 (impulsiveness). Although healthcare users in strata 1 and 2 can be classified as being in lower need of mental care, it is essential assessing the answers given to violence-related items. Table 9 shows the frequency of (‘no’) answers per MHCN stratum for items with the highest β coefficients.

**Table 9:** Frequency of answers per MHCN stratum for items with the highest β coefficients.

	Low MHCN	Moderate MHCN	High MHCN	Very high MHCN
V6. Are you able to keep working?	95.3% (n = 464)	75.4% (n = 159)	66.3% (n = 69)	44.4% (n = 36)
V11. Are you able to control your impulsiveness?	95.5% (n = 465)	81.5% (n = 172)	66.3% (n = 69)	39.5% (n = 32)
V18. Was the user a witness of violence?	9.4% (n = 46)	33.2% (n = 70)	46.2% (n = 48)	51.9% (n = 42)
V20. Was the user a victim of violence?	4.1% (n = 20)	28.9% (n = 61)	39.4% (n = 41)	49.4% (n = 40)

MHCN: mental health care needs. Note: frequency refers to ‘no’ answers.

Next, we examined the scores separately for the eight dimensions, two blocks, and the instrument’s total score. The results show that the score range for all dimensions was equal to the maximum score possible for the dimension, which may indicate the possibility that the instrument is capturing nuances of the information provided by the healthcare user and the health professional. However, block 1 (14 of a maximum possible score of 17), block 2 (13 of a maximum possible score of 14), and the instrument’s total score (19 of a maximum possible score of 31) did not reach their full amplitude (Table 10).

**Table 10:** Median, minimum, maximum, range, and interquartile range of scores for the eight dimensions, two blocks, and the instrument’s total score.

	Median	Minimum	Maximum	Range	Interquartile range
Dimension 1: Social relationships	3	0	3	3	0
Dimension 2: Functioning	4	0	4	4	0
Dimension 3: Autonomy	3	0	3	3	0
Dimension 4: Impulsiveness and aggressiveness	3	0	3	3	0
Dimension 5: Spirituality	4	0	4	4	0
Dimension 6: Violence	0	0	3	3	1
Dimension 7: Self-aggression and suicidal behavior	0	0	7	7	0
Dimension 8: Caregiving plan	0	0	4	4	0
Total Block 1: Self-referred domain	1	0	14	14	2
Total Block 2: Health professional evaluation domain	0	0	13	13	1
Total Instrument	1	0	19	19	3

The analyses enabled the development and validation of an instrument with stable and reliable evidence of internal structure validity as well as classification and interpretation limits sensitive to healthcare users’ different MHCN in PHC settings.

We clarify that CuidaSM was translated from Portugues to English for scientific dissemination purposes, but it has not undergone a translation process with cultural adaptation, meaning that results presented at this study refers to the original version of the scale, available in Portuguese.

## Discussion

The CuidaSM scale developed here show evidence of content and internal structure validity in a PHC setting. The scale includes eight dimensions, of which five were self-referred by healthcare users (Social relationships, Functioning, Autonomy, Impulsiveness and Aggressiveness, and Spirituality) and three were evaluated by PHC professionals (Violence, Self-aggression and Suicidal Behavior, and Caregiving Plan), comprising 31 items distributed into the self-referred block (17 items) and PHC professional evaluation block (14 items).

Scales are developed to measure a phenomenon highlighted by a theory or concept that points towards its existence but that cannot be directly measurable. To achieve that, the idea is to identify factors related to a given latent variable, which provides a reasonably accurate way to measure the phenomenon [59].

MHCN is a complex latent variable. The literature describes a series of factors related to this variable, including socioeconomic, clinical, and disability factors [12]. Other aspects, such as experiencing insecurity and hopelessness, fast social changes, risk of violence, and physical illnesses, are related to higher vulnerability to common mental disorders of people in poverty situations, especially in low and middle-income countries (LMIC) like Brazil [60].

Brazil Ministry of Health (MS) technical recommendations on mental health care suggest some scales to support PCH users’ tracking and/or monitoring [61], depending on the assessed condition, such as Patient Health Questionnaire (PHQ-9) for Depression [62], Clinical Dementia Rating Scale [63], Overall Anxiety Severity and Impairment Scale (OASIS) [64], and CAGE (Cut, Annoyed, Guilty, and Eye) questionnaire [65] and Alcohol Use Disorders Identification Test (AUDIT) for alcohol abuse [66]. However, none of these scales are indicated to support stepped-care decisions. Moreover, it is important to highlight that these scales focus on quantifying symptoms and, in general, do not include the individuals’ perception about mental disorder impairments.

The literature shows that functional impairment and how it is perceived by the individual could indicate where care should be provided within the care provision network [12,67]. The Diagnostic and Statistical Manual of Mental Disorders (DSM-5) diagnostic handbook highlights that functioning assessment should be performed independently of diagnostic considerations and recommends the World Health Organization Disability Assessment Schedule 2.0 (WHODAS 2.0) [68] for assessment of global functioning and impairment.

The CuidaSM scale validated in the current study resembles some WHODAS 2.0 domains, seeing that both were developed based on the International Classification of Functioning, Disability, and Health (ICF) [29]. Moreover, both scales aim at identifying healthcare aspects, establishing priorities, and helping improve resource allocation regardless of disorder etiology. However, although WHODAS 2.0 items were developed to directly correspond to ICF disability dimensions, the CuidaSM scale did not show the same direct correspondence, including other aspects related to the MHCN construct such as Spirituality, Impulsiveness and Aggressiveness, Violence, Self-Aggression and Suicidal Behavior, and Caregiving Plan. Dimension “Functioning” in CuidaSM, which assesses the difficulty dealing with daily activities including those associated with domestic duties, work, and attending health appointments, shows similarities with Domain 5 (Life Activities) in WHODAS 2.0 [68], the items “d850 remunerated job; d830 Higher education; d220 be multi-task” in ICF [29], and the Work and Social Adjustment Scale (WSAS) [69]. Results for this dimension in our sample show that being unable to work had a high frequency of ‘yes’ answers regardless of the MHCN stratum. This finding indicates that item 6, “Are you able to keep working?”, deserves closer attention and must be considered a marker for MHCN regardless of the instrument’s final score.

Dimension “Autonomy” resembles Domain 3 (Self-care) in WHODAS 2.0 [68], and items “d510–d650 combination of multiple self-care factors and domestic life tasks” in ICF [29]. This dimension is relevant because the main focus when seeking to improve individual functioning is ensuring individuals have autonomy to perform practical daily tasks.

The “Social Relationships” dimension of CuidaSM resembles Domain 4 (Relationship) in WHODAS 2.0 and the “Social and Interpersonal Functioning” domain in ICF [29], assessing interactions with other people and difficulties that can be faced because of a health condition. Items in this CuidaSM dimension also resemble WSAS [69], which is used to measure functioning losses due to health issues. The three scales assess the existence of social relationships and the ability to keep them. It is known that social support improves the sense of self-efficacy, leading to increased understanding, respect, encouragement, and self-realization, which can help individuals to keep their emotions relatively stable, even if they are under distress [70].

Even though impulsiveness and aggressiveness symptoms are assessed as part of a broader psychopathology [71–73] and can be observed in almost all psychiatric disorders and in some neurological or clinical diseases [74], they are not classical psychiatric diagnoses such as schizophrenia, depression, bipolar disorder, or personality disorder. In CuidaSM, dimension “Impulsiveness and Aggressiveness” highlights that the presence of these symptoms can be associated with suicidal tendencies [75–76] and impair treatment [77], and is indicative of MHCN. It is worth noting that a high frequency of participants in the current study reported being unable to control their impulsiveness regardless of MHCN stratum. Thus, item 11, “Are you able to control your impulsiveness?”, must also be considered a marker that needs attention regardless of the instrument’s final score.

Dimension “Spirituality” of CuidaSM is comparable to the quality-of-life Spirituality, Religiosity and Personal Beliefs field-test instrument developed by the World Health Organization (WHOQOL-SRPB): both instruments assess how personal beliefs can serve as a coping strategy to deal with issues by giving a meaning to human behavior and by influencing quality of life [78]. Other instruments are available in the literature that measure spirituality [79–85]. Some of these scales deeply explore religion and religiosity [85], whereas others measure well-being and inner peace [84] or feelings such as forgiveness [82] and gratitude [80]. This conceptual multiplicity encompasses the diversity of spirituality as it is understood. Dimension “Spirituality” in the CuidaSM scale views the concept as referring to transcendence, to the sacred, to aspects of life that gain spiritual character and meaning, that give meaning to life, that are related to the observation of beauty and nature, and to the generation of well-being [78].

Results for the CuidaSM “Violence” dimension are consistent with evidence showing an association among violence, poverty, and mental disorders, especially in countries with high social inequality such as Brazil [86]. In our study, 51.9% of participants classified as having very high MHCN gave ‘yes’ answers to item 18, “Was the user a witness of violence?”, whereas only 9.4% of respondents with low MHCN reported having witnessed violence (item 20). Thus, these items can be considered MHCN boosters and deserve special attention when a ‘yes’ answer is given, even if the total score result is low-to-moderate MHCN.

Dimension “Self-Aggression and Suicidal Behavior” is essential to assess MHCN because mental disorders are linked to most cases of suicide [87]. Several scales exist aimed at tracking suicidal behaviors and identifying self-aggression [88–90]. However, suicide is a complex and multicausal phenomenon that requires more accurate tools that go beyond identifying the will to die, suicidal thoughts and plans. In this context, CuidaSM provides an assessment that considers the continuum of suicidal behaviors and self-aggression, contributing to a broader evaluation of MHCN.

Although CuidaSM items and dimensions share some similarities with the instruments discussed above, it is important to highlight that CuidaSM was designed to support professional decision-making within a stepped-care process (based on Model of Care for Chronic Conditions) and not as a screening and/or diagnostic instrument. Thus, CuidaSM is suggested to be administered primarily to healthcare users previously screened for some mental health condition. Considering the invisibility of mental health issues, especially in the Brazilian PHC context, and the high burden of mental disorders, CuidaSM could not only provide a classification for MHCN but also help to expand the professional’s repertoire for profiling population mental health needs.

In this context, dimension “Caregiving Plan”, designed to support professional decision-making about delivering the right care in the right place at the right time, is innovative. Accordingly, scale item 28, “Does the PHC team have a hard time handling this case?”, opens up the possibility for PHC professionals to indicate they may be in need of consulting with an expert, showing the potential for the instrument to support shared healthcare decision-making. This item complies with the collaborative caregiving model (‘matriciamento’ in Portuguese) in Brazil, which recommends PHC professionals should request specialist support when needed to approach a complex case [91].

To strengthen the discussion on the matrix support model, it is important to highlight the recent reintroduction of multiprofessional teams in Primary Health Care, established by Ordinance GM/MS No. 635 on May 22, 2023 [92]. These teams are fundamental for the effective use of the scale, ensuring comprehensive care and fostering collaboration in the creation and implementation of individualized care plans, thus enhancing patient outcomes and integrated service delivery.

Brazil Ministry of Health [61] technical recommendations on mental health care represent an improvement because they seek to systematize the offer of mental health actions across the public health system, i.e.,:

*“describe routines of patients’ routes, complete information about the promotion, prevention, treatment and rehabilitation actions, and activities to be developed by a multi-disciplinary team in each healthcare service; they make the communication feasible among teams, services, and user within a Healthcare Assistance Network, with emphasis on action standardization, by organizing an assistance continuum.”* (Brazil Ministry of Health, 2022)

According to the Model of Care for Chronic Conditions [26], these actions are offered based on population needs and clinical guidelines. This way of organizing the delivery of care is similar to the approach taken for stepped-care protocols [27–28], which are a sequential approach to healthcare whereby the majority of patients have access to low-intensity treatments that offer less restrictive, cheaper interventions for most individuals; patients who remain symptomatic can then have access to more intensive and costly therapies.

Another approach to scaling healthcare consists of determining the patients eligible for low- or high-intensity therapies by stratifying patients based on an initial evaluation. Stratified care was more effective and cost-effective to treat depression symptoms than stepped care in a recent randomized clinical trial study [93]. By stratifying MHCN, the CuidaSM scale can provide objective information to support decision-making processes regarding the best location for delivering treatment to an individual [94–95]. Accordingly, by stratifying individuals with MHCN, CuidaSM has the potential to support PHC professionals in choosing the most adequate interventions for a patient. Thus, CuidaSM contributes to the rational use of technical and human resources by allocating them according to groups with different MHCN.

CuidaSM has the potential to standardize healthcare-sharing criteria among different services within the care provision network; thus, it potentially supports joint planning of healthcare delivery among different attention levels by informing joint decision-making processes and promoting collaborative care. It is worth highlighting that CuidaSM integrates different dimensions into a single comprehensive instrument, simplifying and accelerating the assessment of MHCN, and facilitating its implementation in clinical practice.

Systematized identification of people with mental health conditions in the study setting is lacking. Thus, a limitation of this study is that data collection was conducted with healthcare users who seek the PHC service for any number of health problems, including but not limited to individuals with risk factors for mental disorders and/or previous diagnosis of mental illness. Even though this may have affected the scale score, it suggests that the instrument may be applicable to a broader population. Thus, future studies should be conducted with populations with previously identified MHCN.

## Conclusion

The numerous strategies used to provide evidence of content and internal structure validity show that the CuidaSM scale can be considered a valid, adequate, reliable, and consistent instrument capable of assessing MHCN and supporting Brazilian PHC professionals to face decision-making-related challenges in mental health care practice. The analysis of a multiregional and multicultural heterogeneous population allows that extrapolation of the results to the national level. From this perspective, CuidaSM is a valid tool that can support PHC teams by standardizing care-sharing criteria within the network, fostering collaborative decision-making and integrated care. With the potential to optimize the use of technical and human resources by allocating them according to different mental healthcare needs, we hope that CuidaSM can contribute to efforts to reduce the mental healthcare gap in Brazil.
